# Lacerated Wound of Tongue: Amputation

**Published:** 1881-07

**Authors:** 


II.—LACERATED WOUND OF TONGUE—AMPUTATION.—Dr. Walton was called to see the horse February 12th, 1881. The tongue was protruding from the mouth, greatly swollen, and quite cold and blue. During the night of the 10th the animal had run his tongue through a hole in the stall, and a neighboring horse had bitten it. It was also supposed to have been frozen subsequently. The tongue for the first two days was washed with a solution of zinc, and allowed to remain hanging out in the cold.

The horse entered the hospital on the 12th, in the above-described condition. The tongue, although cold to the touch, was thought worth trying to save. At this time the horse had lost all power to draw it into the mouth. The tongue was carefully cleansed with warm water. It was placed in a bag, and gently pushed into the mouth, and retained in position by tapes passing from the bag over the head back of the ears. It was hoped that by sustaining it in position the horse would regain the power to hold it within the mouth, and that the coldness and discoloration would be overcome by the warmth, and gangrene prevented. The mouth was washed with carbolic acid lotion, and a weak solution of liq. sodii chlorinatis; but on the second day it became more swollen, the discharge from the mouth grew very offensive, and gangrene seemed inevitable. The tongue was therefore removed on the fifth day after the receipt of the injury. A cord was drawn tightly around the organ, about two inches posterior to the discolored portion, and the tongue divided in the sound tissue with one sweep of the knife. Two hours after its removal the cord was removed. No marked hemorrhage occurred, though blood oozed from the stump for four hours after. There was no secondary hemorrhage, and the wound healed quite rapidly, so that the animal was ready to work in three days after the excision was performed. The only after treatment was a cleansing of the parts three times daily.

